# Revisiting the Idea That Amyloid-β Peptide Acts as an Agonist for P2X7

**DOI:** 10.3389/fnmol.2020.00166

**Published:** 2020-09-17

**Authors:** Lučka Bibič, Leanne Stokes

**Affiliations:** School of Pharmacy, University of East Anglia, Norwich, United Kingdom

**Keywords:** Alzheimer disease, P2X7 receptor, microglia, reproducibility, amyloid beta peptide

## Abstract

The P2X7 receptor (P2X7) is a cell surface ligand-gated ion channel, activated by its physiological nucleotide agonist ATP and a synthetic analog (BzATP). However, it has also been suggested that there may be structurally unrelated, non-nucleotide agonists such as the amyloidogenic β peptide. Here we aimed to reassess the effect of amyloid β peptides in various *in vitro* cell models, namely HEK293 overexpressing human P2X7, the microglial BV-2 cell line, and BV-2 cells lacking P2X7. We measured YO-PRO-1 dye uptake in response to full-length amyloid β peptide (1–42) or the shorter amyloid β peptide (25–35) and there was a concentration-dependent increase in YO-PRO-1 dye uptake in HEK-hP2X7 cells. However, these amyloid β peptide-induced increases in YO-PRO-1 dye uptake were also identical in non-transfected HEK-293 cells. We could observe small transient increases in [Ca^2+^]_*i*_ induced by amyloid β peptides in BV-2 cells, however these were identical in BV-2 cells lacking P2X7. Furthermore, our metabolic viability and LDH release experiments suggest no significant change in viability or cell membrane damage in HEK-hP2X7 cells. In the BV-2 cells we found that high concentrations of amyloid β peptides (1–42) and (25–35) could reduce cell viability by up to 35% but this was also seen in BV-2 cells lacking P2X7. We found no evidence of LDH release by amyloid β peptides. In summary, we found no evidence that amyloid β peptides act as agonists of P2X7 in our *in vitro* models. Our study raises the possibility that amyloid β peptides simply mimic features of P2X7 activation.

## Introduction

The most prevalent cause of dementia is Alzheimer’s disease, a fatal neurodegenerative disorder that is characterized by a progressive cognitive and functional impairment and memory loss (Heppner et al., 2015). In the field of Alzheimer’s disease research, the amyloid cascade hypothesis has been the major hallmark of pathogenesis. This states that the generation of amyloid plaques, primarily composed of the amyloid-β peptide (Aβ), represents the initial event triggering neurobiological dysfunction (Hardy and Selkoe, 2002). Over two decades of research (Hardy and Higgins, 1992) have recently revealed many layers of complexity (Lee et al., 2018), however, the bulk of data still supports the role of the Aβ peptide as the primary initiator of Alzheimer’s disease pathogenesis (Musiek and Holtzman, 2015; Cheignon et al., 2018).

Previous data have indicated that the immune system may have a role in Alzheimer’s disease and that activated microglia have been observed in patients (Sarlus and Heneka, 2017). Microglia, the resident macrophages of the CNS, alter their morphology and phenotype to adopt a so-called activated state in response to neurophysiological brain insults (Heneka et al., 2015; Sarlus and Heneka, 2017). Morphologically activated microglia are believed to contribute to the progression of Alzheimer’s disease via receptors such as the scavenger receptor CD36 (El Khoury et al., 1996), α6β1 integrins (Koenigsknecht and Landreth, 2004), the formyl peptide receptor-like protein (Le et al., 2001), TLR2 (Chen et al., 2006), TLR4 (Michaud et al., 2013) and the TLR-interacting molecule CD14 (Fassbender et al., 2004). As a result, this leads to inflammatory mediator secretion and microglial responsiveness to injury as comprehensively reviewed by Sarlus and Heneka (2017). However, another receptor that is also highly expressed by microglial cells is P2X7, an ATP-gated ion channel (Chessell et al., 1997; Ferrari et al., 1997; Bartlett et al., 2013). In recent years, several studies suggested the participation of P2X7 in Aβ-mediated brain damage (Haughey and Mattson, 2003; Parvathenani et al., 2003; Rampe et al., 2004; McLarnon et al., 2006). Furthermore, Sanz et al. (2009) suggested that P2X7 may participate in microglia activation by Aβ peptides and it has since been proposed that ATP might not be the only endogenous agonist for P2X7 receptors (Di Virgilio et al., 2018). This may, in turn, open new avenues for the development of novel therapies for Alzheimer’s disease. Intrigued by this proposal of alternative agonists, we set out to determine if Aβ could act as a P2X7 agonist and investigate the effect of positive allosteric modulators of P2X7 that we have previously characterized (Helliwell et al., 2015). However, we have been unable to validate the results that suggest that Aβ-induced responses require the expression of P2X7. Based on our data, we conclude that Aβ peptides may not directly cause P2X7-dependent signaling in microglial cells.

## Materials and Methods

### Cell Culture

Microglial BV-2 and BV-2 P2X7-deficient cells were maintained in DMEM/F12 with L-glutamine (Gibco 11320-074, Fisher Scientific, United Kingdom), containing 10% (v/v) FBS (Gibco, US origin), penicillin and streptomycin (Fisher Scientific, United Kingdom). HEK-293 cells were maintained under the same media conditions. HEK-293 cells stably expressing human P2X7 were generated previously (Bhaskaracharya et al., 2014) and maintained under similar conditions with the addition of 400 μg/ml geneticin (Fisher Scientific, United Kingdom). All cells were maintained at 37°C with 5% CO_2_ in a humidified incubator.

### Materials

Aβ peptides corresponding to human Aβ amino acids Aβ_25__–__35_, Aβ_35__–__25_ (inactive scrambled peptide) and Aβ_1__–__42_ were purchased from GenScript, United States and prepared as 10 mM stock solutions in either water or DMSO. Apyrase (an ATP-hydrolyzing enzyme), ATP and BzATP were purchased from Sigma Aldrich, United Kingdom. Stock solutions of P2X7 antagonists AZ10606120 and JNJ47965567 (Tocris Bioscience, Bio-Techne, United Kingdom) were prepared in DMSO (10 mM) and stocks were kept frozen at −20°C.

### Measurements of Intracellular Ca^2+^

The cells were seeded at 2 × 10^5^ cells/well (100 μl) and plated on poly-D-Lysine coated 96-well plates (Nunc 167008, Fisher Scientific, United Kingdom) and used for experiments 24 h after plating. Cells were loaded with the indicator dye Fura-2-AM using a concentration of 2 μM (HelloBio, United Kingdom) in HBSS for 45 min at 37°C. Loading buffer was removed and replaced with 180 μl of assay buffer containing 147 mM NaCl, 2 mM KCl, 0.1 mM CaCl_2_, 13 mM Glucose, 10 mM HEPES; pH 7.35. The plate was warmed in the FlexStation 3 (Molecular Devices, United Kingdom) for 10 min before recording using excitation wavelengths 340 and 380 nm and emission at 520 nm (Bibic et al., 2019).

Agonists (10x concentration) were automatically injected at 30 s using the Flex function. Data was converted to Fura-2 ratio (340/380) and normalized using a zero baseline correction. Area under the curve was calculated and plotted. All data was collected in triplicate.

### Measurements of YO-PRO-1 Dye Uptake

Impermeant dye uptake was measured with the extracellular fluorescent tracer YO-PRO-1, a probe that enters the cells through P2X7 activation-induced pores and emits fluorescence when it binds DNA (Idziorek et al., 1995). A solution of 2 μM YO-PRO-1 in assay buffer (see above) was added to wells, and the 96-well plate was placed at 37°C for 10 min. The fluorescence signal in response to drug injection was then measured using a Flexstation 3 (Molecular Devices) as described previously (Bidula et al., 2019; Dhuna et al., 2019). Excitation wavelength was 490 nm and emission was measured at 520 nm. Machine settings include bottom read fluorescence, PMT medium, 6 reads/well with a sample interval of 3.5 s. RFU data was normalized using a zero baseline correction and area under the curve was calculated and plotted. All data was collected from triplicate wells in each independent experiment.

### Cell Viability Measurements

Cells were plated at 2 × 10^4^/well (in a volume of 100 μl) and plated on non-coated 96-well plates (Fisher Scientific, United Kingdom) in culture medium containing 1% FBS 24 h before stimulation. Stimuli included concentrations of Aβ peptides (10–60 μM), ATP at 500 μM and 3 mM concentrations, as well as staurosporine (5 μM), and the vehicle control (DMSO). Following incubation with different stimuli, resazurin (0.1 mg/ml in PBS, Sigma Aldrich) was added to cells for final 2 h at 37°C. The plate was then read on a Flexstation 3 plate reader using an excitation wavelength of 570 nm and emission wavelength of 600 nm (Bidula et al., 2019).

### LDH Release Assay

Lactate dehydrogenase (LDH) release into cell culture supernatants was measured using an LDH assay kit (Pierce, Fisher Scientific, United Kingdom) following the manufacturer’s instructions. Control cells were lysed with the lysis buffer provided to harvest total intracellular LDH. For measuring LDH release, cells were cultured in 96-well plates, stimuli applied for 24 h, and supernatants were collected. Absorbance of duplicate 50 μl aliquots of supernatants were measured on a Flexstation 3 plate reader at 490 nm.

### Data Analysis and Statistics

All results are expressed as mean ± SD using data from collated experiments. All experimental data was collected from triplicate wells. All data for cell viability and cytotoxicity were obtained as relative fluorescence units (RFU) and are expressed as a percentage of the negative control (culture medium). Statistical analysis was performed using one-way ANOVA followed by Tukey’s multiple comparison *post-hoc* test (GraphPad Prism v8). Statistically significant differences from controls are indicated by ^∗^ using *p* < 0.05 as a threshold.

## Results

**FIGURE 1:**
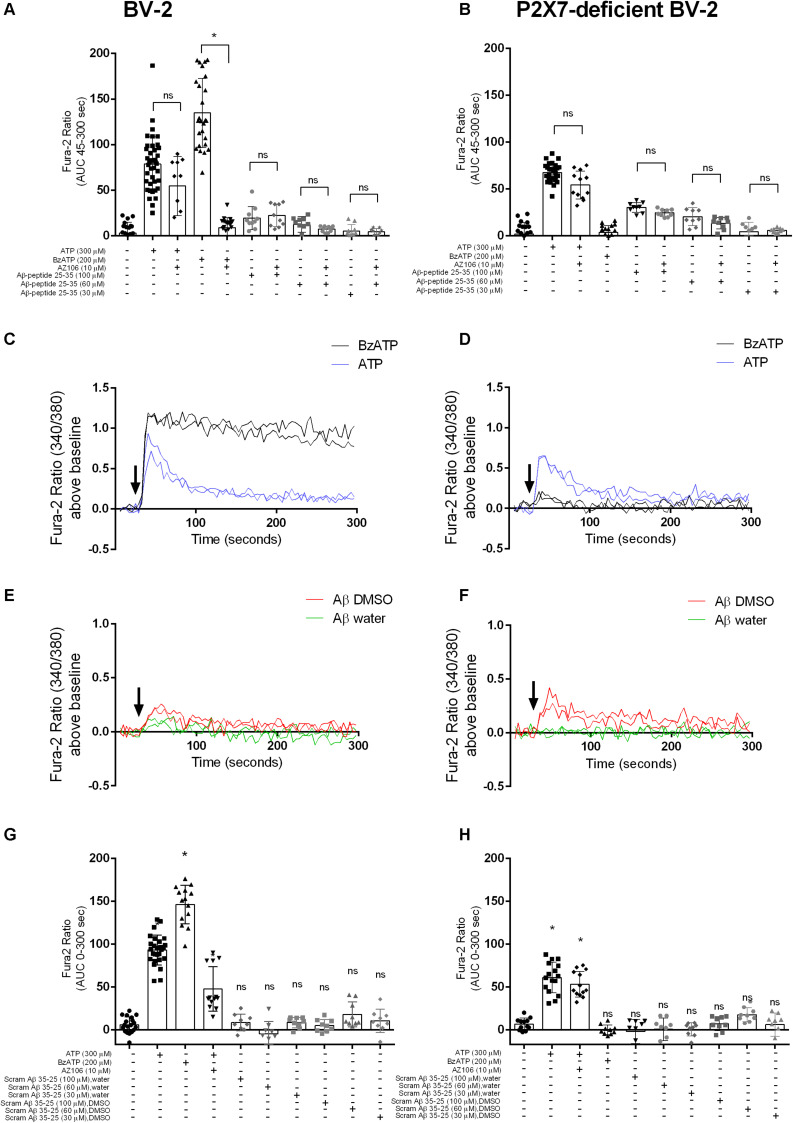
Aβ_25__–__35_ induced similar [Ca^2+^]i responses in microglial BV-2 cells in the presence/absence of P2X7. BV-2 cells were loaded with Fura-2AM (2 μM) and challenged with the Aβ_25__–__35_ peptides (DMSO) in the concentration range 30–100 μM in either BV-2 **(A)** or P2X7-deficient BV-2 **(B)**. One-way ANOVA was performed with Tukey’s multiple comparison test where * indicates a significant difference to the paired control (with AZ1060610) and ns denotes no significant difference. Each symbol represents a single well and data has been collated from all independent experiments. Kinetics of the [Ca^2+^]i response in either BV-2 **(C,E)** or P2X7-deficient BV-2 **(D,F)** are plotted from a representative experiment. Similarly, BV-2 cells were loaded with Fura-2AM (2 μM) and challenged with the inactive scrambled Aβ_35__–__25_ peptide in the concentration range 30 μM -100 μM in either BV-2 **(G)** or P2X7-deficient BV-2 **(H)**. AZ10606120 is a selective antagonist of P2X7, and BzATP is a synthetic agonist for P2X7 receptors. Experiments were repeated five times with triplicates on each plate, and the results are presented as area under curve (mean ± SD). One-way ANOVA was performed with Tukey’s multiple comparison test where * indicates a significant difference (*P* < 0.05) to the control (buffer alone) and ns denotes no significant difference.

A study using N13 microglial cells indicated that Aβ peptide, both the full-length 1–42 and the shorter 25–35, may induce responses similar to the activation of P2X7 (Sanz et al., 2009). We show that stimulation of BV-2 microglial cells with ATP and BzATP, both known agonists for P2X7, led to [Ca^2+^]_*i*_ increases (Figures 1A,C). Using a P2X7-deficient BV-2 cell line generated by CRISPR/Cas9 gene editing (Dhuna et al., 2019), the [Ca^2+^]_*i*_ responses were different to the parental BV-2 line, with the response to BzATP almost completely abolished (Figures 1B,D). We first tested Aβ_25__–__35_ and found that this peptide did induce a transient increase in [Ca^2+^]_*i*_ increase in BV-2 cells (Figures 1A,E) that displayed a concentration-dependent effect. We observed a similar Aβ_25__–__35_ -induced [Ca^2+^]_*i*_ increase in the P2X7-deficient BV-2 cells (Figures 1B,F). Furthermore, we show that the Aβ_25__–__35_ induced [Ca^2+^]_*i*_ increases were not affected by the P2X7-selective antagonist AZ10606120 whereas the ATP-induced [Ca^2+^]_*i*_ increase was reduced 30% by AZ10606120 (Figure 1A) in BV-2 cells. As expected, there was no significant effect of AZ10606120 in P2X7-deficient BV-2 cells (Figure 1B). The inactive scrambled Aβ_35__–__25_ peptide did not increase [Ca^2+^]_*i*_ to the same level as Aβ_25__–__35_ in BV-2 microglial cells, whether expressing P2X7 (Figure 1G) or not (Figure 1H). Some evidence suggests that the solvent, such as DMSO, acetonitrile, and water, may influence the self-assembly and thus the biological activity of Aβ peptides (Busciglio et al., 1992; Shen and Murphy, 1995; Zagorski et al., 1999). In our experiments Aβ_25__–__35_ was prepared in either DMSO or water (Figure 1) and peptide dissolved in DMSO displayed higher Aβ_25__–__35_ -induced [Ca^2+^]_*i*_ increases (Figures 1E,F and Supplementary Figure S1).

**FIGURE 2:**
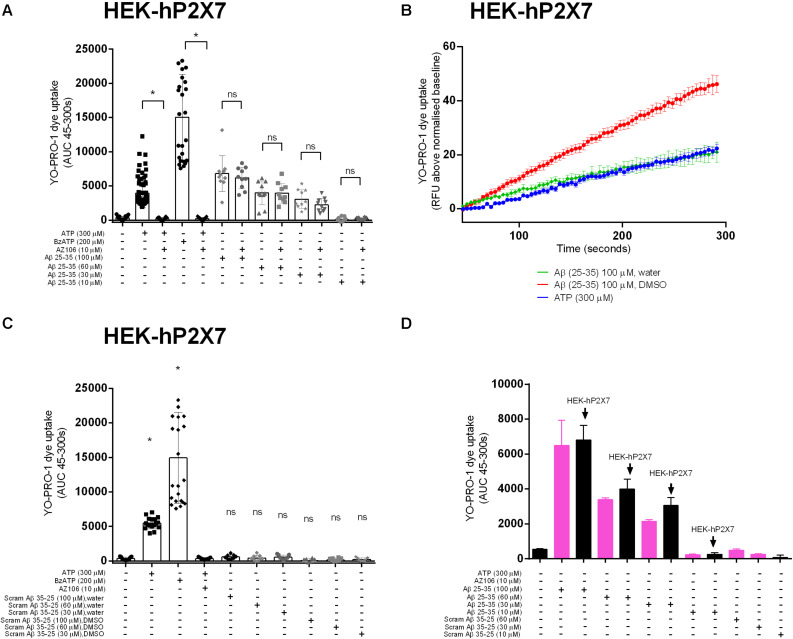
Aβ peptides triggered a non-specific YO-PRO-1 uptake in HEK-hP2X7 and HEK-293 cells. HEK-hP2X7 and plain HEK-293 cells were incubated with YO-PRO-1 dye in low-divalent assay buffer (2 μM) and challenged with either Aβ_25__–__35_ **(A,B)** or the inactive scrambled version Aβ_35__–__25_ **(C)**. Kinetics of the Aβ_25__–__35_ dye uptake response in HEK-hP2X7 are plotted together with the appropriate controls **(B)**. AZ10606120 is a selective antagonist of hP2X7, and BzATP is a synthetic agonist for hP2X7 receptors. **(D)** shows summary data from HEK-293 cells comparing responses to HEK-hP2X7 cells. Experiments were repeated five times with triplicates on each plate, and the results are presented as mean ± SD. One-way ANOVA was performed with Tukey’s multiple comparison test where * indicates a significant difference (*P* < 0.05) to the paired control [with AZ1060610 in **(A)** or control in **(C)**] and ns denotes no significant difference.

We next tested whether Aβ_25__–__35_ would induce responses in a stable HEK-293 cell line over-expressing human P2X7 (HEK-hP2X7). We used a standard YO-PRO-1 dye uptake assay to assess the P2X7 large pore formation (Bibic et al., 2019). We observed that Aβ_25__–__35_ (Figures 2A,B), but not the inactive scrambled Aβ_3__5__–__25_ (Figure 2C), induced a significant YO-PRO-1 uptake over the concentration range 30–100 μM. Notably, this Aβ_25__–__35_-induced dye uptake was not abrogated by the P2X7-specific antagonist AZ10606120 despite complete blockade of ATP- and BzATP-induced dye uptake in this cell line (Figure 2A). Furthermore, Aβ_25__–__35_-induced dye uptake was not affected by the ATP-degrading enzyme apyrase (Supplementary Figure S2). In addition, we observed that Aβ_25__–__35_ induced YO-PRO-1 dye uptake into non-transfected HEK-293 cells which fail to display ATP-induced dye uptake (Figure 2D) supporting the hypothesis that the observed effects of Aβ_25__–__35_ are not dependent on the expression of P2X7. The inactive scrambled Aβ_35__–__25_-peptide did not induce YO-PRO-1 dye uptake responses (Figures 2B,C). The Aβ_25__–__35_-induced dye uptake was affected by the solvent used with the DMSO-peptide having a greater effect than the water-solubilized peptide (Figure 2B and Supplementary Figure S2).

**FIGURE 3:**
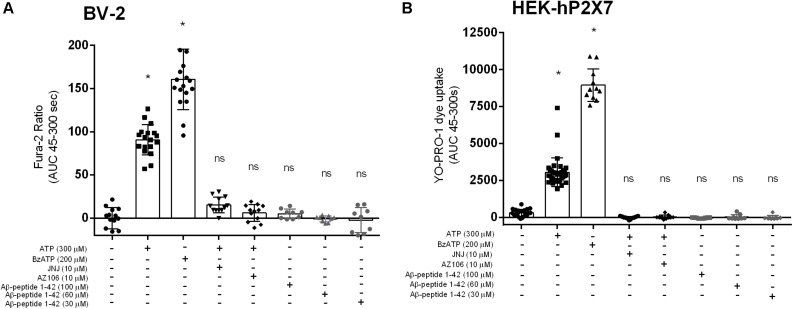
Aβ_1__–__42_ do not trigger responses in BV-2 cells or HEK-hP2X7 cells. BV-2 cells were loaded with Fura-2AM (2 μM) and challenged with the Aβ_–__1__–__42_ **(A)** peptides in the concentration range 30–100 μM. HEK-hP2X7 cells were exposed to YO-PRO-1 dye in low-divalent assay buffer (2 μM) and challenged similarly as BV-2 cells **(B)**. AZ10606120 is a commercially available antagonist of hP2X7. Experiments were repeated five times with triplicates on each plates, and the results are presented as mean ± SD. One-way ANOVA was performed with Tukey’s multiple comparison test where * indicates a significant difference (*P* < 0.05) to the control (buffer alone) and ns denotes no significant difference.

We investigated the full-length Aβ peptide, 1–42, which was also reported by Sanz et al. (2009) to have an effect on microglial IL-1β secretion. We did not observe [Ca^2+^]_*i*_ increases in BV-2 (Figure 3A) or YO-PRO-1 dye uptake in HEK-hP2X7 (Figure 3B) in response to Aβ_1__–__42_ peptide at concentrations of 10, 60 or 100 μM. Both ATP and BzATP induced robust responses in both cell lines and the P2X7-selective antagonists AZ10606120 and JNJ47965567 could abolish these responses (Figure 3). Even at the highest concentration of Aβ_1__–__42_, this peptide did not cause a significant increase in [Ca^2+^]_*i*_ release relative to the buffer control.

**FIGURE 4:**
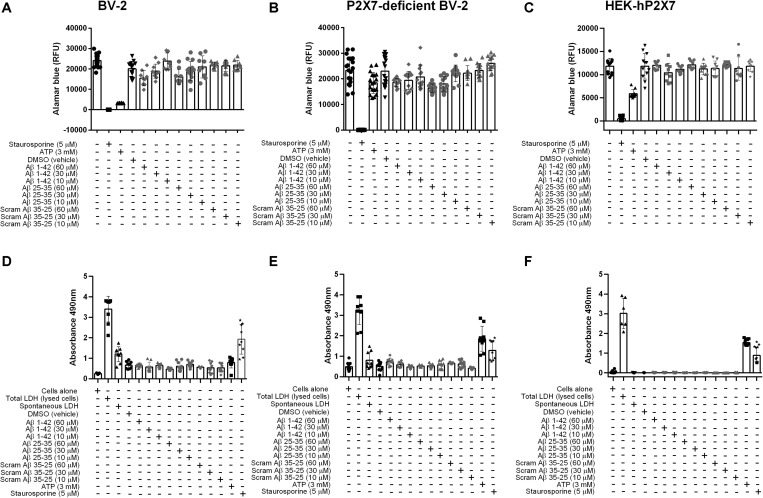
Aβ peptides do not trigger P2X7-specific cell death nor compromise the cell membrane in microglial BV-2 cells or HEK-hP2X7 cells. **(A–C)** An AlamarBlue assay and **(D–F)** an LDH assay were performed to assess cell viability and cell death respectively after 24 h administration of Aβ peptides. At indicated times (see “Materials and Methods”), the extracellular medium was collected and assayed for lactate dehydrogenase (LDH) activity. Control cells were lysed with the lysis buffer to induce maximal LDH release for normalization of the LDH release (% of max). Staurosporine and 3 mM ATP were used as a positive control. Data points represent the mean ± SD of 5 replicated experiments with triplicates on each plate. One-way ANOVA was performed with Dunnett’s multiple comparison test where * indicates a significant difference (*P* < 0.05) to the control (media alone).

Once activated, P2X7 is endowed with the ability to kill microglia, either by necrosis or apoptosis, as well as to trigger many responses such as inflammation and oxidative stress (Surprenant et al., 1996; Ferrari et al., 1997; Brough et al., 2002; Volonte et al., 2012). Thus, we examined whether Aβ_25__–__35_ or Aβ_1__–__42_ could trigger cell death in BV-2, P2X7-deficient BV-2 cells, or HEK-hP2X7 cells at either 10, 30 or 60 μM, using a cell viability assay (Figure 4). In BV-2 (Figure 4A) and P2X7-deficient BV-2 cells (Figure 4B), Aβ_1__–__42_ or Aβ_25__–__35_ (60 μM) reduced cell viability similarly up to 35 and 30% of control, respectively, suggesting that this effect was not P2X7 dependent. Lower concentrations of Aβ_1__–__42_ or Aβ_25__–__35_ (10, 30 μM) had lesser effects and no significant decrease in cell viability was found with Aβ_1__–__42_ or Aβ_25__–__35_ on HEK-hP2X7 cells (Figure 4C). The inactive scrambled Aβ_35__–__25_ peptide did not have any effect on the cell viability (Figures 4A–C). Cell supernatants were also measured for the presence of LDH, which is released upon cell lysis. Figures 4D–F shows LDH levels from BV-2 cells, P2X7-deficient BV-2 cells, and HEK-hP2X7 cells. None of the Aβ-treated HEK-hP2X7 cell supernatants contained significant levels of released LDH as compared with control samples of cells stimulated with staurosporine or 3 mM ATP. In the BV-2 cells and P2X7-deficient BV-2 cells, we found a relatively high spontaneous LDH release and none of the Aβ peptide treatments were higher than this (Figures 4D,E).

## Discussion

ATP is a crucial extracellular messenger serving as the physiological agonist of both P2Y and P2X receptors (Wang et al., 2004; Davalos et al., 2005; Haynes et al., 2006; Koizumi et al., 2007). The identification of alternative agonists for P2X receptors is of particular interest when ascribing physiological roles to individual receptors in different cells and tissues. There has been a suggestion that ATP may not be the only agonist at P2X7 receptors (Di Virgilio et al., 2018). It is proposed that agents such as amyloid–β (Sanz et al., 2009), serum amyloid (Niemi et al., 2011) and the cathelicidin LL-37 peptide (Elssner et al., 2004), may function as non-nucleotide agonists at P2X7. In the current study, we revisited the role of the amyloid-β peptide as an agonist for the P2X7 receptor using a microglial cell line and HEK-293 cells over-expressing human P2X7. We have previously used the microglial BV-2 cells and a clonal P2X7-deficient BV-2 cell line, generated using Cas9 gene editing, in order to assess Ca^2+^ influx upon P2X7 activation with ATP and the effect of positive modulators (Dhuna et al., 2019).

The biological effect of synthetic amyloid-β peptides, including Aβ_25__–__35_, may vary due to differences in aggregation states (Pike et al., 1993; Wei and Shea, 2006), therefore we performed the experiments using two common solvents, DMSO (Mattson et al., 1993) and water (Whitson et al., 1994). Our data shows that Aβ_25__–__35_ directly induced intracellular Ca^2+^ responses in BV-2 microglial cells regardless of the solvent (Figure 1) although responses were noted to be higher for the DMSO-dissolved peptide. We saw no increase in [Ca^2+^]_*i*_ to the full length human Aβ_1__–__42_ peptide. Furthermore, when these two amyloid-β peptides (Aβ_25__–__35_ and Aβ_1__–__42_) were studied in a HEK-hP2X7 stable cell line using a dye uptake assay (Helliwell et al., 2015; Bibic et al., 2019; Bidula et al., 2019; Dhuna et al., 2019), we observed some YO-PRO-1 dye uptake in response to Aβ_25__–__35_. However, this also occurred in non-transfected HEK-293 cells that do not express P2X7 receptors (Figure 2). We saw no increase in YO-PRO-1 dye uptake to the full length human Aβ_1__–__42_ peptide. Collectively this data suggests that amyloid-β peptides may act on other receptors that can induce similar responses to P2X7 or the peptide may act by insertion into the lipid bilayer forming similar pores. It is known that other agents such as maitotoxin and ionomycin can induce dye uptake responses to P2X7 stimulation (Schilling et al., 1999; Verhoef et al., 2004) likely through insertion into the membrane and subsequent pore formation. There is evidence that amyloid-β peptides may act via other routes such as on pannexin-1 (Orellana et al., 2011) to cause ATP release from cells rather than acting to directly activate P2X7. This was also suggested to be the likely (indirect) effect on microglial cells in the work by Sanz et al. (2009). We did not directly measure ATP release in our study but we hypothesized that any ATP released by the amyloid-β peptides would elicit responses at P2X receptors and therefore we would have seen an effect of the P2X7-selective antagonists, apyrase, or in the BV-2 cells with P2X7 deficiency.

Human and rodent (rat/mouse) full length amyloid-β peptide (1–42) are highly similar and differ only in three amino acid substitutions at the N-terminus. It is not clear which isoform of the Aβ_1__–__42_ was used in the study by Sanz et al. (2009) however, we believe this minor sequence difference is unlikely to contribute to our lack of effect at P2X7. Indeed, should there be a species difference, we would expect to see responses at human P2X7 (which we did not). The amino acid sequence for Aβ_25__–__35_ peptide is identical between human and rodent. In our study we focused on addressing whether the amyloid-β peptides could act as agonists at P2X7. We did not extend our experiments to investigate the effect of amyloid-β peptides on IL-1β secretion from microglial cells. Gustin et al. demonstrated that Aβ_25__–__35_ could induce IL-1β secretion from LPS-primed mouse microglia but did not detect any ATP secretion in response to Aβ_25__–__35_. Furthermore, this team of researchers used primary microglia from the P2X7^–/–^ mouse and observed a similar IL- 1β secretion in response to Aβ_25__–__35_ (Gustin et al., 2015). This notion led them to conclude that Aβ_25__–__35_-induced IL-1β secretion was P2X7-independent (Gustin et al., 2015). This contradicts the earlier work from Sanz et al. who demonstrated that amyloid peptides could induce IL-1β release from primary microglia but only when P2X7 was present (Sanz et al., 2009). Furthermore, *in vivo* evidence showed that amyloid β-induced IL-1β secretion in the hippocampus was reduced in P2X7 knockout mice (Sanz et al., 2009). Other studies have shown that activation of microglia with Aβ_1__–__42_, followed by exposure to BzATP, may result in enhanced secretion of IL-1β (Rampe et al., 2004). This suggests that amyloid-β peptides may be working as positive allosteric modulators rather than as agonists. Furthermore, McLarnon et al. demonstrated that Ca^2+^ responses in adult microglial cells from Alzheimer’s disease patients were significantly increased following Aβ_1__–__42_ pre-treatment when activated by a P2X7 selective agonist BzATP (McLarnon et al., 2006). We have tested the idea that amyloid-β peptides could act as positive allosteric modulators since this was our entry point into this set of experiments. However, we saw no enhancement of ATP-induced [Ca^2+^]_*i*_ responses by Aβ_1__–__42_ peptide and no potentiation of ATP-induced YO-PRO-1 dye uptake in HEK-hP2X7 cells (Supplementary Figure S3).

Microglial cell death (measured as a reduction in cellular viability) can be observed when cells were stimulated by 3 mM ATP (Nishida et al., 2012; Dhuna et al., 2019). When the BV-2 cells were stimulated by either Aβ_1__–__42_, Aβ_25__–__35_ or the inactive scrambled Aβ_35__–__25_ peptide (60 μM), microglial cell viability was reduced by 35% compared to control. There was no significant increase in LDH release above vehicle treatment or spontaneous LDH release induced by water. Our data indicate that Aβ peptides may not act as non-nucleotide agonists of the P2X7 receptor and that Aβ peptides are unable to induce cytotoxicity or decrease cell viability directly via P2X7. We are not discarding P2X7 as an emerging therapeutic target for Alzheimer’s disease. Others have shown that P2X7 is involved in amyloid protein precursor (APP) processing (Delarasse et al., 2011) and affects phagocytosis of Aβ peptides (Ni et al., 2013). More recently, the P2X7 knockout mouse was investigated in the APPPS1 mouse model of Alzheimer’s disease (Martin et al., 2019). P2X7^–/–^mice had a reduced amyloid-β load and were protected from cognitive defects. In this model the chemokines CCL3, CCL4, and CCL5 were elevated only when P2X7 was present and this affected CD8^+^ T cell recruitment to the choroid plexus and hippocampus (Martin et al., 2019). It appears that there is still more to be discovered about how P2X7 can influence the development of neurodegenerative disorders.

## Data Availability Statement

The datasets generated for this study are available on request to the corresponding author.

## Author Contributions

LB conceived and designed the study together with LS. LB performed the experiments and analyzed the data. LS and LB wrote the manuscript and edited the final version. Both authors contributed to the article and approved the submitted version.

## Conflict of Interest

The authors declare that the research was conducted in the absence of any commercial or financial relationships that could be construed as a potential conflict of interest.
