# Supplementary material for: Revisiting the Idea That Amyloid-β Peptide Acts as an Agonist for P2X7
Source: Front Mol Neurosci. 2020 Sep 17;13:166. doi: 10.3389/fnmol.2020.00166 (PMC7530339; doi:10.3389/fnmol.2020.00166)
Supplement: Supplementary file 1 [file Table_1.docx]

Supplementary Material

Revisiting the idea that amyloid beta peptide acts as an agonist for P2X7

Lučka Bibič, Leanne Stokes*

*Corresponding Author: Leanne Stokes
e-mail: [l.stokes@uea.ac.uk](mailto:l.stokes@uea.ac.uk)

This docx. file includes:

Figs. S1 to S3

**A**

**B**

**Figure S1.** **Aβ_25-35_ induced [Ca^2+^]_i_ responses in microglial BV-2 cells** **when the Aβ_25-35_ peptides are dissolved in water.** (A) BV-2 cells and (B) P2X7-deficient BV-2 cells were incubated in Ca^2+^ containing buffer (see Methods) and challenged with the Aβ_25-35_ peptide, dissolved in water, and in the concentration range 30µM -100µM. AZ10606120 is a commercially available antagonist of hP2X7, and BzATP is a specific agonist for P2X7 receptors. Data points represent the mean ± SD of 5 replicated experiments with triplicates on each plate.

**Figure S2.** **Aβ_25-35_ and YO-PRO-1 dye uptake in HEK293-hP2X7 cells when the Aβ_25-35_ peptides are dissolved in water.** HEK293-hP2X7 cells were incubated with 2 µM YO-PRO-1 dye in low-divalent buffer (see Methods) and challenged with Aβ_25-35_ peptides dissolved in water over the concentration range 30µM -100µM. AZ10606120 is a commercially available antagonist of hP2X7, and BzATP is a specific agonist for P2X7 receptors. Apyrase was used to metabolise any released ATP in the media. Data points represent the mean ± SD of 5 replicated experiments with triplicates on each plate.

**Figure S3. Aβ_1-42_ does not potentiate ATP-induced YO-PRO-1 uptake in HEK293-hP2X7 cells** Cells were incubated with YO-PRO-1 dye in the low-divalent buffer (see Methods) and were pre-incubated with the Aβ_1-42_ peptide in the concentration range 30µM – 30nM. AZ10606120 and JNJ (JNJ47965567) are the commercially available antagonists of hP2X7, and ATP is an agonist for P2X7 receptors. Data points represent the mean ± SD of 5 replicated experiments with triplicates on each plate. One-way ANOVA was performed with Dunnett’s multiple comparisons test using 1 mM ATP as the control, * represent P<0.05.
